# Posterior Reversible Encephalopathy Syndrome in a Late Postpartum Patient With a Rare Complication of Subarachnoid Hemorrhage

**DOI:** 10.7759/cureus.56042

**Published:** 2024-03-12

**Authors:** Zara H Siddiqui, Justin G Hovey, James S Bolton

**Affiliations:** 1 Obstetrics and Gynecology, Alabama College of Osteopathic Medicine, Dothan, USA; 2 Internal Medicine/Pediatrics, Alabama College of Osteopathic Medicine, Dothan, USA; 3 Internal Medicine/Pediatrics, Southeast Health Medical Center, Dothan, USA; 4 Radiology, Southeast Health Medical Center, Dothan, USA

**Keywords:** spontaneous subarachnoid hemorrhage, hypertensive disorders of pregnancy (hdp), postpartum eclampsia, postpartum preeclampsia, posterior reversible encephalopathy syndrome (pres)

## Abstract

Posterior reversible encephalopathy syndrome (PRES) is considered a neuroclinical syndrome of headache, confusion, visual changes, and seizures associated with neuroimaging findings of posterior cerebral white matter edema. Although the incidence of the syndrome is largely unknown, this condition is becoming increasingly recognized. The prognosis is generally good with most symptoms resolving within one week and lesions on imaging resolving in two weeks. Death and significant neurological disability have been reported but are relatively rare. In this report, we present a 10-day postpartum patient with an atypical history of headache and seizure-like activity. Neuroimaging revealed findings consistent with PRES as well as a rare complication of subarachnoid hemorrhage. This case highlights the importance of clinicians considering preeclampsia/eclampsia-induced PRES when encountering a postpartum patient with headache and hypertension to further reduce morbidity and mortality in this patient population.

## Introduction

Posterior reversible encephalopathy syndrome (PRES) was first described in the literature as posterior reversible leukoencephalopathy syndrome in 1996 [1]. It is a neuroclinical syndrome that is associated with seizures, visual disturbances, headache, altered mental status, and/or focal neurological deficits [2]. There are a multitude of causes of PRES including preeclampsia, eclampsia, (acute) severe hypertension, renal failure, immunosuppression, autoimmune diseases, and infection; however, the pathophysiology remains unclear. The incidence of PRES is unknown, but there are numerous documented case studies in the medical literature. Magnetic resonance imaging (MRI) of the brain is the gold standard for diagnosis and commonly reveals bilateral symmetric hyperintensities in the parietal and occipital regions on T2 fluid-attenuated inversion recovery (FLAIR) images. Clinical symptoms of PRES are reversible, and most patients’ symptoms resolve within one week. The MRI findings typically resolve more slowly over weeks. Although specific treatment guidelines do not exist, antihypertensives, antiepileptics, withdrawing potentially triggering drugs, and treating the underlying disease(s) are generally accepted [2].

Preeclampsia and eclampsia complicate 2-8% of pregnancies worldwide and are commonly reported causes of PRES, but the incidence in this specific patient population is unknown [3, 4]. In developed countries, 16% of maternal deaths are attributed to hypertensive disorders [5]. Typically, preeclampsia is diagnosed based on specific criteria for hypertension and proteinuria. Eclampsia is diagnosed when there is a new-onset seizure activity before, during, or after labor in the setting of preeclampsia [3]. Interestingly, 20-38% of women do not present with signs of preeclampsia prior to developing seizures [3]. The timing of eclampsia diagnosis may be shifted due to the increased screening and treatment of preeclampsia during the antenatal period [6]. This change in timing does pose a risk for specific populations. Clinicians and pregnant/postpartum women unaware of the risk may ignore the signs or not seek care for their symptoms [6, 7]. Furthermore, a diagnosis of late postpartum preeclampsia or eclampsia can be considered if the patient develops signs and symptoms greater than 48 hours but less than four weeks after delivery [8, 9]. Approximately 16% of eclampsia diagnoses can be categorized as late postpartum eclampsia [7, 9]. Here, we report a 19-year-old, 10-day postpartum female who presented with atypical headache and seizure-like activity. She was diagnosed with PRES and had a prolonged hospital course. Ultimately, her symptoms and neuroimaging findings resolved. This case report discusses PRES pathophysiology, clinical findings, association with preeclampsia/eclampsia, management, and key takeaways for clinicians.

## Case presentation

A 19-year-old G1P1001 female with a significant past medical history of childhood migraines and spontaneous vaginal delivery occurring 10 days prior presented to the clinic of her primary care provider with a complaint of intractable headache. The patient was primigravida, and she denied any complications during the pregnancy and delivery. In fact, she was discharged home three days postdelivery. The patient described her headache as progressively worsening over a four-day period with severe stabbing pain behind the left eye. The headache progressed to “all over” the head at the time of the presentation, and she described feeling as if her head was “going to explode.” She rated the pain as 10/10 in severity. She reported associated photophobia, phonophobia, nausea, and visual changes including blurred vision. The patient also reported sharp, nonradiating right upper quadrant (RUQ) pain since her delivery that she rated as 6/10 in severity. Vital signs at this visit were a temperature of 97.9°F, heart rate of 121 bpm, blood pressure of 122/89 mmHg, and respiratory rate of 16 breaths per minute. The pain began two days after delivery, was intermittent, and resolved about six days after delivery. She was given abortive migraine therapy at the clinic and was scheduled for an outpatient MRI the next day.

When the patient did not get any relief from the abortive migraine medication and over-the-counter analgesics four hours after leaving the physician’s office, she was instructed to go to the emergency department (ED) immediately. When the patient first arrived at the ED with her mother, she had seizure-like activity outside the entrance of the hospital. A second episode of seizure-like activity occurred in triage with the triage staff describing her eyes rolling back into her head with jerking movements that lasted about one minute. She had no urinary or fecal incontinence.

The patient had no previous medical history of seizures, hypertension, or gestational hypertension. Her medications included cetirizine 10 mg oral tablet daily and an iron supplement 45 mg oral tablet daily. Her only previous surgery was an uncomplicated cholecystectomy for cholelithiasis. She had no previous hospitalizations. She has no known allergies. She denied any alcohol, tobacco, or illicit drug use. The patient denied any significant contributory family history including neurologic or vascular disorders.

Her initial vital signs upon admission were a blood pressure of 158/73 mmHg, pulse of 157 bpm, temperature of 101.3°F, respiratory rate of 22 breaths per minute, and O_2_ saturation of 97%. A physical exam revealed the patient in moderate distress with sonorous respirations. Examination of the mouth revealed no lingual lacerations. Cardiovascular exam revealed sinus tachycardia with normal rhythms without murmurs, rubs, or gallops. No peripheral edema was noted. Respiratory exam revealed normal respiratory effort, and lungs were clear to auscultation bilaterally. Genitourinary exam revealed minimal dark lochia. The abdomen was soft, nontender, and without hepatosplenomegaly. Neurological exam revealed no focal deficits with intact and symmetric deep tendon reflexes without clonus. Gag reflex and corneal reflexes were intact. Glasgow Coma Scale (GCS) score was 12.

Initial laboratory findings in the ED revealed complete blood count and peripheral blood smear within normal limits with the exception of white blood cell (WBC) count of 36,300 mL and platelet count of 834,000 mL. Other abnormal lab values of note were a lactate of >20 mmol/L and bicarbonate of 10 mmol/L. Antinuclear antibody (ANA) testing was positive (see Table 1). No cerebrospinal fluid was obtained. Initial chest X-Ray showed no acute cardiopulmonary process. 

**Table 1:** Patient Lab Values Abbreviations: WBC (white blood cell), AST (alanine transaminase), ALT (aspartate aminotransferase), LDH (lactate dehydrogenase), CRP (C-reactive protein), ESR (erythrocyte sedimentation rate), TSH (thyroid stimulating hormone), PT (prothrombin time), PTT (partial thromboplastin time), INR (international normalized ratio), TIBC (total iron-binding capacity), UIBC (unsaturated iron-binding capacity), ANA (antinuclear antibody)

Lab	Patient’s value	Reference range
WBC	36,300 (80% segmented neutrophils with 3% bands)	4500-11,000/mL
Platelets	834,000	150,000-450,000/mL
Creatinine	0.6	0.5-1.10 mg/dL (female)
AST	15	10-40 units/L
ALT	10	10-40 units/L
Alkaline phosphatase	215	30-120 units/L
Creatinine kinase	89	30-135 units/L (female)
Lactate	>20	6.3-18.9 mg/dL
Potassium	3.1	3.5-5.0 mEq/L
Magnesium	1.9	1.6-2.6 mEq/L
Glucose	161	<140 mg/dL
Bicarbonate	10	23-28 mEq/L
LDH	323	80-225 units/L
CRP	2.12	0.8 mg/dL
ESR	30	0-20 mm/hour
Fibrinogen	346	200-400 mg/dL
TSH	0.92	0.5-4.0 microunits/mL
Vitamin D	14.19	15-60 pg/mL
Hemoglobin A1c	5.4%	4-5.6%
PT	12.7	11-13 seconds
PTT	23.7	25-35 seconds
INR	1.03	<1.1
TIBC	435	250-310 microg/dL
Iron % saturation	10%	15-55%
UIBC	390	131-425 mmol/L
Urinalysis	0 protein	0 protein
ANA	positive	negative

The patient met the systemic inflammatory response syndrome (SIRS) criteria on admission with objective symptoms of fever, tachycardia, and leukocytosis present. There was no obvious source of infection, so blood cultures were obtained, and she was subsequently started on meropenem and vancomycin for broad spectrum antibiotic coverage.

After evaluation and stabilization, the patient was sent for a stat CT (computed tomography) of the head without contrast, where she had a third episode of witnessed seizure-like activity. At that time, she was noted to be hypertensive with a blood pressure of 188/120 mmHg and a heart rate of 165 bpm. She was immediately started on intravenous (IV) levetiracetam, IV magnesium, IV nifedipine, and IV fluids. 

**Figure 1:**
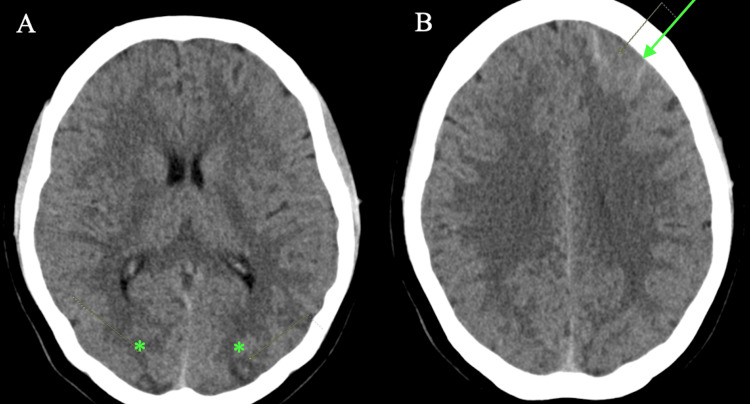
Noncontrast Brain CT (1A) Noncontrast CT indicating low-density areas in the cortical and subcortical region of both occipital lobes (*). (1B) Vague edema and minimal subarachnoid blood in the left frontal lobe (arrowhead)

CT of the head showed low-density areas in the cortical and subcortical regions of both the occipital lobes and vague edema and suspected minimal subarachnoid blood in the left frontal lobe (Figures 1A-1B). The frontal lobe subarachnoid hemorrhage was confirmed on MRI as well.

Subsequent CT of the abdomen and pelvis revealed no acute process. It showed normal appendix, slightly distended bladder, postgravid uterus with appropriate fluid and air in the endometrial canal, and gallbladder surgically absent with no ductal dilation.

Upon returning to the ED, neurosurgery was consulted and requested MRI, magnetic resonance venography (MRV), and magnetic resonance angiography (MRA) of the brain to rule out intracerebral venous thrombosis. While waiting to obtain an MRI, the patient became more agitated, confused, and unresponsive to IV diazepam. Initially, the patient was able to protect her airway and follow some commands but waned significantly while awaiting the MRI. She was subsequently intubated.

**Figure 2:**
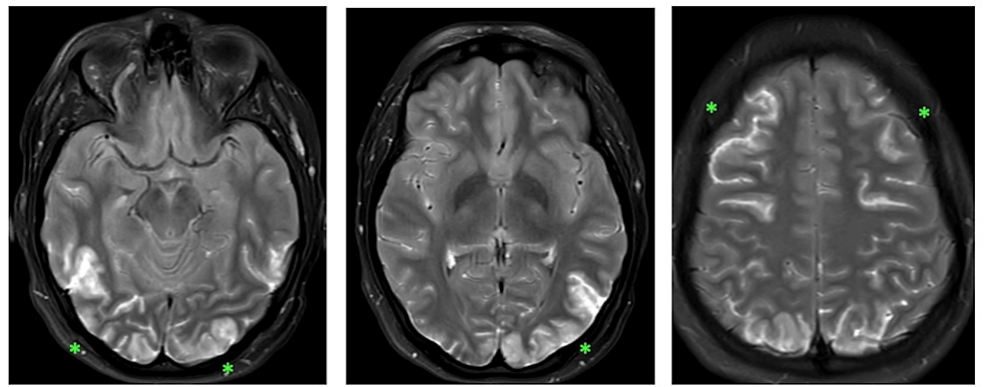
Axial T2-Weighted MRI MRI (magnetic resonance imaging). Bilateral near-symmetrical areas of increased signal abnormality in the frontal and occipital regions (*).

MRI showed scattered bilateral and symmetric areas of cortical edema on axial T2 imaging involving predominantly the occipital lobes and to a lesser extent the frontal lobes (Figure 2). There was no parenchymal hematoma, no hydrocephalus, and no extra-axial fluid collection. MRA was negative and showed no evidence of arterial thrombosis, stenoses, or aneurysm. MRV showed normal venous sinuses, without evidence of venous sinus or cortical vein thrombosis. There were no focal areas of restricted diffusion seen indicating that there was no infarct or permanent damage.

Once intracerebral thrombosis was ruled out, neurosurgery suggested no surgical intervention and recommended mannitol 12.5 g IV which was started promptly. A consultation with the neurology service recommended the addition of dexamethasone 8 mg IV TID for the vasogenic edema as well as continuation of levetiracetam. The patient was subsequently admitted to the neurological intensive care unit (ICU) for further monitoring and treatment.

The patient’s clinical status improved on day two of admission, and she was extubated. A repeat CT of the head showed improvement of the hypodense changes in the bilateral posterior cerebral hemispheres with mild residual as well as resolution of the subarachnoid bleed in the left anterior frontal lobe. Her clinical status continued to improve, and she was transferred out of the neurological ICU on day three. Two peripheral blood cultures showed no growth in five days. The patient’s WBC count decreased to 9.1. The patient was clinically and hemodynamically stable on day five, and she was discharged home at that time on levetiracetam, dexamethasone, and nifedipine. 

The patient was seen in the clinic for follow-up one month after discharge. She reported intermittent headaches since discharge. She stated the pain behind her left eye was not as severe as it was prior to admission. Her blood pressure was well controlled on nifedipine. She was still taking levetiracetam but completed her course of dexamethasone. Repeat MRI of the brain showed complete resolution of her previous findings.

## Discussion

Given this patient’s presentation including headache, hypertension, seizures, and altered mental status, cerebral venous thrombosis, ischemic stroke, and cerebral hemorrhage needed to be ruled out first. MRI was negative for cerebral venous thrombosis and ischemic stroke but did suggest the presence of a small-volume subarachnoid hemorrhage. In addition to PRES, the differential diagnosis of a postpartum patient with neurological symptoms includes reversible cerebral vasoconstriction syndrome (RCVS), which is another disorder of cerebral arterial dysfunction with clinical features that can include sudden headaches, visual symptoms, and seizures. However, the patient’s four-day history of progressively worsening headache rather than a sudden headache makes this diagnosis less likely and was ultimately ruled out based on the lack of vasospasm on MRA. In addition, given the patient's history of childhood migraines, a migraine headache with aura leading to seizure was also on the differential. This was also less likely due to CT and MRI findings which were consistent with PRES.

Hypertensive encephalopathy is another major etiology on the differential which can be characterized by acute and severe elevation of blood pressure (>180/120 mmHg) leading to cerebral edema and neurologic symptoms. These patients usually have insidious onset of headache which can progress to seizures and often hypertensive retinopathy [10]. There is a significant overlap between hypertensive encephalopathy and PRES, but given the lack of chronic severe hypertension in this patient and the presence of classic bilateral occipital edema, PRES is a more likely diagnosis.

Another differential diagnosis in this scenario included infectious etiology due to the patient meeting the criteria of systemic inflammatory immune response (SIRS) given fever, tachycardia, elevated WBC count, and elevated lactate. For example, there has been one case report of PRES associated with retained products of conception [11]. However, given our patient’s lack of lower abdominal pain or postpartum hemorrhage with a benign-appearing postgravid uterus and appropriate fluid and air seen on CT imaging, this was also ruled out. The patient’s negative blood cultures and negative urine analysis suggest that her fever, elevated WBC count, thrombocytosis, elevated lactate, elevated CRP, and sedimentation rate were reactive in nature rather than an indicator of sepsis. Identification of an autoimmune disease process was not pursued on this admission and could not be ruled out completely given the positive ANA levels. A positive ANA has low specificity and could be detected for a number of reasons including low vitamin D levels, connective tissue diseases, and a normal postviral immune response even in healthy subjects. Overall, a positive ANA does not have a diagnostic value [12].

Thrombosis to the patient’s portal or splanchnic circulation was considered due to the patient’s RUQ pain, elevated alkaline phosphatase, and preeclampsia/eclampsia, but without clear evidence and no acute process seen on CT abdomen/pelvis imaging, this was low on the differential. The patient’s RUQ pain points to eclampsia as a cause or contributing factor in the development of PRES, considering 25% of eclamptic patients present with RUQ pain [13]. Additionally, Hemolysis, elevated liver enzymes, and low platelet (HELLP) syndrome was ruled out given the lack of evidence of hemolysis, elevated liver enzymes, or thrombocytopenia.

The patient’s symptomology in addition to the classic findings of bilateral and symmetric cortical edema on MRI predominantly in the occipital lobes but also the frontal lobes led to a diagnosis of PRES. The loss of signal in the sulci of the left frontal lobe suggestive of minimal subarachnoid hemorrhage most likely represented a PRES-induced subarachnoid hemorrhage.

The pathophysiology of PRES is not fully understood, but it is generally considered to be a dysregulation of cerebral perfusion [14]. Although there are different theories surrounding the mechanism of perfusion pressure change, the shared outcome involves the breakdown of blood-brain barrier with extravasation of fluid, plasma proteins, and blood [15]. It is generally accepted that this vasogenic edema causes the hyperintensities seen in neuroimaging [16]. Moreover, the posterior regions of the brain are more susceptible to impaired autoregulation because there is relatively less sympathetic innervation to the posterior circulation [15]. There are two major theories discussed regarding how perfusion pressure changes in the brain: the hyperperfusion theory and the hypoperfusion theory.

The hyperperfusion theory could explain the cases of hypertension-associated PRES, which makes up approximately 70% of cases [17]. It postulates that rapid increases in arterial pressure cause cerebral hyperperfusion. This increase in blood pressure overwhelms the autoregulatory capabilities of the vessels, leading to arterial expansion, endothelial injury, and excessive perfusion. The severe vasodilation results in the breakdown of the blood-brain barrier and the accumulation of vasogenic edema. While this theory is the most popular, some authors have questioned whether hypertension in PRES patients is elevated enough to truly alter autoregulation and disrupt the vasculature, as approximately 30% of cases reveal normotensive patients [17, 18].

The hypoperfusion theory is considered in cases of PRES triggered by immunosuppressive therapy, sepsis, autoimmune diseases, and preeclampsia/eclampsia. This mechanism postulates that specific circulating toxins cause endothelial activation and injury [2]. The endothelial injury leads to focal cerebral vasospasm; the vasospasm causes decreased cerebral perfusion, ischemia, and subsequent vasogenic edema and cytotoxic edema [2, 7]. Preeclampsia is generally considered to be the result of defective remodeling of the placenta as it invades the uterine wall [19]. The disordered growth causes placental ischemia, and subsequently, fetal debris enters the maternal circulation. There have been documented changes in circulating levels of pro-angiogenic and antiangiogenic factors as well as the release of inflammatory cytokines such as tumor necrosis factor alpha (TNF-α), interleukin-1 (IL-1), interferon gamma (IFNg), and interleukin-6 (IL-6) [7, 20]. This triggers a maternal systemic immune response leading to widespread endothelial cell activation and injury. The activated cells release inflammatory mediators and vasoconstrictors while decreasing circulating vasodilators resulting in systemic vascular contraction [7, 20]. The endothelial cell activation and injury also cause hemolysis and platelet adhesion leading to thrombocytopenia, elevated lactate dehydrogenase (LDH), the presence of schistocytes on peripheral blood smear, renal dysfunction, and edema [7]. Our patient’s elevated LDH and thrombocytosis support this process, but she lacked additional evidence of hemolysis.

The most common signs and symptoms of PRES are seizures, headaches, visual disturbances, and altered mental status. Seizures are the most frequent and usually the first symptom of PRES [2]. Most patient’s symptoms progressively worsen over a 12-to-24-hour period [2]. This case is distinct from usual presentations because our patient reported a four-day history of worsening headaches prior to developing seizures on the fourth day in the ED.

Preeclampsia and eclampsia are the most common triggers of PRES, in addition to hypertensive encephalopathy, decreased renal function, immunosuppressive therapy, autoimmune diseases, and sepsis [2, 7, 15]. Preeclampsia can be defined as the new onset of systolic blood pressure of >140 mmHg and/or diastolic blood pressure of >90 mmHg after 20 weeks gestation in addition to proteinuria. Our patient did meet the criteria for preeclampsia with severe features based on the definition from the American College of Obstetricians and Gynecologists (ACOG): hypertension, severe persistent RUQ pain, and new-onset headache [3]. Eclampsia can be diagnosed in a patient with preeclampsia or another hypertensive disorder of pregnancy that has a generalized seizure that is not attributed to other causes [3]. It can also occur in the postpartum period, as with the patient in this case, in up to 20% of individuals [13].

The incidence of PRES in the preeclampsia and eclampsia patient population is unknown [21]. However, there have been numerous small studies demonstrating that the majority of patients with preeclampsia and eclampsia reveals findings consistent with PRES on neuroimaging. A study by Chao et al. revealed 12 of 20 preeclamptic patients had abnormal hyperintensities in the cortical areas of the occipital and temporal lobes [22]. Brewer et al. found radiologic evidence of PRES in 46 of 47 eclamptic patients [23]. Mayama et al. found 12 of 13 patients with eclampsia and five of 26 patients with preeclampsia had MRI findings consistent with PRES [24]. In a study by Zeeman et al., 25 of 27 (93%) nulliparous women with eclampsia had reversible vasogenic edema on diffusion-weighted MRI [25]. Given the evidence of close association, PRES and eclampsia may be clinically difficult to distinguish and may even represent the same physiologic process. These findings suggest that it is important for clinicians to have a low threshold for imaging when encountering a patient before, during, or after labor with signs and symptoms of preeclampsia or eclampsia. Increased recognition and diagnosis of PRES will lead to prompt treatment and subsequently decrease the risk of PRES-associated complications including cerebral ischemia and intracranial hemorrhage.

The radiologic findings of PRES are characterized by white matter vasogenic edema involving the parietal and occipital lobes bilaterally and symmetrically [2, 15, 16]. Common additional findings include involvement of the frontal lobes, especially near the superior frontal sulci [15]. Complications of PRES such as subarachnoid hemorrhage are less common; some authors indicate intracranial hemorrhage has been identified in 15-19% of cases, 4-30% of cases, or 9-33% of cases [2, 15, 16, 26]. In a retrospective study of 263 patients with PRES, 51 of these patients presented with a related intracranial hemorrhage [27]. Of these patients, 46% had an intraparenchymal hemorrhage, and 14% had a subarachnoid hemorrhage. It was observed that the subarachnoid hemorrhages spared the basal cisterns in all cases and were usually small [27]. Our patient’s imaging revealed a subtle sulcal hyperintensity in the region of the anterior frontal lobe suggesting a small-volume subarachnoid hemorrhage. This minimal sulcal hyperintensity seen on MR imaging suggests that the subarachnoid hemorrhage is most likely a complication of PRES, as opposed to SAH-induced PRES which would present as a diffuse hyperintensity with involvement of the basal cisterns [26]. Developing intracranial hemorrhage as a complication from PRES has impacts on morbidity and mortality [26]. Fortunately, our patient recovered from PRES and the subarachnoid hemorrhage with no permanent neurological deficits.

No randomized controlled trials involving the treatment of PRES have been performed. Anecdotally, the crucial elements of management in PRES are seizure control and lowering of blood pressure after ensuring airway patency and adequate oxygenation [2, 21]. It is also important to address the underlying trigger of PRES on a case-by-case basis, such as withdrawing immunosuppressive medications or controlling hypertension. In the case of preeclampsia or eclampsia, treating seizures with magnesium sulfate is necessary as well as the delivery of the fetus if symptoms occur in the antenatal period. If seizures remain uncontrolled, benzodiazepines or phenytoin can be used. Antihypertensive options used in this patient population include labetalol, hydralazine, or nifedipine with a treatment goal of <160-140 systolic blood pressure and <110-90 diastolic blood pressure [21].

PRES lesions are reversible; most patients’ symptoms resolve within one week, with the MRI lesions resolving more slowly over days to weeks. Diffusion-weighted MRI from the patient in this case showed that there was no infarct or permanent damage inflicted on the brain, indicating that the process was completely reversible. Although most patient’s lesions reverse over time, there have been cases of permanent neurological damage. This is usually due to the conversion of vasogenic edema to cytotoxic edema [2]. Recurrent seizures and epilepsy are rare, and if patients are discharged on antiepileptics, they are typically tapered after three months of therapy [4]. In a retrospective study involving 78 patients with PRES, there was a reoccurrence rate of 4% [2, 4].

## Conclusions

When considering the two proposed theories for the pathophysiology of posterior reversible encephalopathy syndrome, our patient’s presentation and clinical findings do not fit neatly into either category. There was no evidence of vasoconstriction on MRA and minimal evidence of hemolysis to support the endothelial dysfunction and hypoperfusion theory. When considering the hyperperfusion theory, the patient’s blood pressure was elevated on arrival in the ED and was acutely elevated during a seizure, but these findings could be a complication of PRES rather than the cause. We suspect that a combination of endothelial dysfunction and unrecognized hypertension due to late postpartum preeclampsia led to our patient developing PRES. It is important to remember that 20-38% of eclamptic patients have no signs or symptoms of preeclampsia prior to developing seizures. With new evidence suggesting that any asymptomatic preeclamptic patient may have an MRI consistent with PRES, clinicians should have a high degree of suspicion for late preeclampsia or eclampsia in a postpartum patient presenting with headache and hypertension. There should be a low threshold for ordering neuroimaging on these patients. Only with prompt diagnosis and treatment can we minimize complications of PRES and the long-term morbidity and mortality in this patient population.
